# Quantum Zeno and Zeno-like effects in nitrogen vacancy centers

**DOI:** 10.1038/srep17615

**Published:** 2015-12-01

**Authors:** Jing Qiu, Yang-Yang Wang, Zhang-Qi Yin, Mei Zhang, Qing Ai, Fu-Guo Deng

**Affiliations:** 1 Department of Physics, Applied Optics Beijing Area Major Laboratory, Beijing Normal University, Beijing 100875, China; 2 Center for Quantum Information, Institute for Interdisciplinary Information Sciences, Tsinghua University, Beijing 100084, China

## Abstract

We present a proposal to realize the quantum Zeno effect (QZE) and quantum Zeno-like effect (QZLE) in a proximal ^13^C nuclear spin by controlling a proximal electron spin of a nitrogen vacancy (NV) center. The measurement is performed by applying a microwave pulse to induce the transition between different electronic spin states. Under the practical experimental conditions, our calculations show that there exist both QZE and QZLE in a ^13^C nuclear spin in the vicinity of an NV center.

Quantum Zeno effect^1^,^2^ (QZE) is a very interesting phenomenon in quantum physics, in which the evolution of a quantum system can be inhibited by frequent measurements. In 1990, based on Cook’s theoretical proposal^3^, QZE^4^ was experimentally demonstrated by controlling the transition between two hyperfine levels of ^9^Be^+^ with laser pulses, and there was a good agreement between theoretical prediction and experimental results. Since then, much effort has been paid to the research on QZE^5^,^6^,^7^,^8^, quantum anti-Zeno effect^9^,^10^,^11^,^12^ and quantum Zeno-like effect (QZLE)^13^,^14^. Because of its potential application in slowing down or even freezing the dynamic evolution of a system via repeated frequent measurements, it recently has attracted considerable interest as a tool in the fields of quantum information processing^15^,^16^,^17^,^18^,^19^,^20^,^21^ and ultrasensitive magnetometer^22^.

QZE has been successfully demonstrated on various physical systems, such as trapped ions^23^, superconducting qubits^24^,^25^, cavity quantum electrodynamics^7^,^16^, nuclear magnetic resonance^26^,^27^, and Bose-Einstein condensates^28^,^29^,^30^. On the other hand, in solid-state quantum-information technology, a nitrogen vacancy (NV) center which consists of a nitrogen substituting for a carbon and an adjacent vacancy in diamond has been identified as one of the most promising candidates for qubits^31^,^32^,^33^,^34^,^35^,^36^,^37^,^38^ due to its long coherence time at room temperature^39^,^40^,^41^ and convenient manipulation under optical field, microwave field and rf field^42^,^43^. Quantum Zeno-like phenomenon was experimentally demonstrated by inhibiting coherent spin dynamics induced by the microwave driving between two ground-state electron-spin levels (*m*_*s*_ = 0 and *m*_*s*_ = 1) of a single NV center^44^. Therein, only one measurement is performed to analyze the measurement effect on electron-spin states, i.e., the population variation between the electron-spin states *m*_*s*_ = 0 and *m*_*s*_ = 1 has been made by this single measurement.

In the conventional QZE, repeated instantaneous perfect measurements performed on the system will freeze the evolution of the initial state. The perfect conventional QZE requires infinite measurements with repetitive frequency approaching infinity, which might be impossible in experiment. However, it was recently discovered that perfect freezing of quantum states can also be achieved by more realistic non-projective measurements performed at a finite frequency^13^. According to Brouwer’s fixed-point theorem^45^, there always exist some quantum states which satisfy Φ(*ρ*_0_) = *ρ*_0_, where Φ represents a quantum dynamical evolution process of a system with its initial state *ρ*_0_. After *n* identical cycle process, the system stays at the state as the same as its inital one, i.e., Φ^*n*^(*ρ*_0_) = *ρ*_0_. In this way, a QZLE can be achieved with finite-frequency measurements.

In this paper, inspired by the discovery in ref. 13, we present a proposal to achieve the QZLE in a proximal ^13^C nuclear spin of an NV center by controlling the electron spin. In our proposal, the electron spin plays the role as a detector while the ^13^C nuclear spin acts as the target. Furthermore, the conventional QZE is demonstrated by modulating the measurement parameters and the external magnetic field. Here, instead of projective measurements, we apply a microwave pulse to induce the transition between different electronic states, followed by initialization of electron spin. Our numerical calculation properly shows that for suitable parameters there exist the conventional QZE and QZLE in a proximal ^13^C nuclear spin of the NV center.

## Results

### The model

An NV center in diamond is composed of a nitrogen atom and a vacancy in an adjacent lattice site. It is a defect with C_3v_ symmetry^46^,^47^. For the negatively-charged NV center with electron spin *S* = 1, the ground state is a spin-triplet state ^3^A with a zero-field splitting *D* = 2.87 GHz between spin sublevels *m*_*s*_ = 0 and *m*_*s*_ = ±1^48^. Around NV centers there are three kinds of nuclear spins^49^,^50^, i.e., ^13^C (*I* = 1/2), ^14^N (*I* = 1), and ^15^N (*I* = 1/2). They can be manipulated by microwave and rf fields.

**Figure 1:**
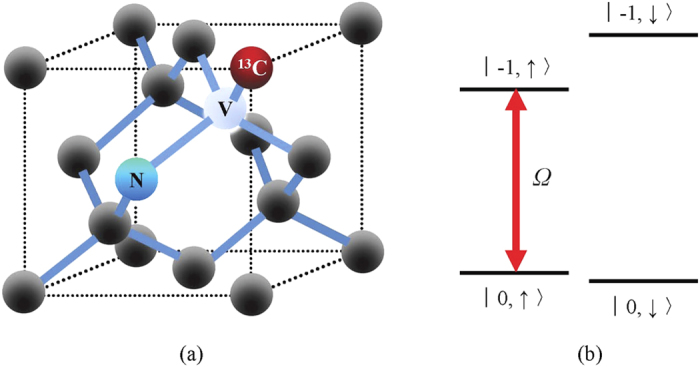
Scheme for demonstration of the QZE in an NV center. (**a**) A ^13^C nuclear spin is at the nearest-neighbor lattice site of an NV center. (**b**) The energy-level diagram of the ground state hyperfine structure where a microwave drives the transition between 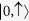 and 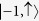 with Rabi frequency Ω and driving frequency *ω*.

Consider an NV center and a ^13^C nuclear spin which locates in the first coordination shell around the NV center^51^, as shown in Fig. 1(a). In other words, this ^13^C nuclear spin is at the nearest-neighbor lattice site of the NV center. As a result, there is a strong hyperfine coupling between the nuclear and electronic spins. Figure 1(b) shows the simplified energy-level diagram of the ground-state hyperfine structure associated with the nearest-neighbor ^13^C nuclear spin. To demonstrate the QZE, the electron-spin states (*m*_*s*_ = −1, 0) and nuclear-spin states 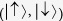 are chosen to code qubits. The target and detector are initially uncorrelated, i.e., they are in a product state. A strong electron-spin polarization into the *m*_*s*_ = 0 sublevel can be induced by circulatory optical excitation-emission. This effect results from spin-selective non-radiative intersystem crossing to a metastable state lying between the ground and excited triplet states^52^,^53^. Moreover, the nuclear spin could be well isolated from the electron spin, during the optical polarization and measurement of the electronic state^42^,^54^. In other words, the state of nuclear spin could be unperturbed when the initialization and measurement are performed on the electronic spin.

Suppose that the electron spin is initially in its ground state 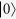 and the nuclear spin is in an arbitrary state. First of all, the whole system evolves freely for a time interval Δ*t*_*f*_. Afterwards, a microwave driving is used to perform measurement. As Fig. 1(b) shows, the microwave drives the transition between 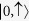 and 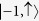 with Rabi frequency Ω and driving frequency *ω*. In the process of measurement, the total system evolves under the Hamiltonian *H*_*M*_ = *H*_*F*_ + *H*_*I*_ for a time interval Δ*t*_*m*_, where *H*_*F*_ is the free Hamiltonian without measurement and *H*_*I*_ is the interaction Hamiltonian describing the transition induced by microwave driving. After the measurement, by optical pumping, the electron spin is initialized in its ground state 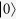, and meanwhile the electron and nuclear spins are decoupled^42^,^54^,^55^. And then the above process is repeated. When the duration of the 532-nm light pulse for optical pumping is appropriate, the ^13^C nuclear spin could be well isolated from the electron spin and the nuclear spin state can be preserved. In particular, the dephasing of nuclear spin can hardly be observed for light pulses of ~140 ns which are sufficiently long to polarize the electron spin while leave the state of nuclear spin undisturbed^42^.

#### The free Hamiltonian H_F_

The general Hamiltonian of an NV center and a ^13^C nuclear spin which locates in the first coordination shell around the NV center is^56^


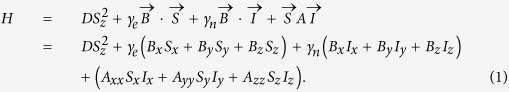


Here, the first term stands for the zero-field splitting of the electronic ground state. The second term is the Zeeman energy splitting of the electron with *γ*_*e*_ being the electronic gyromagnetic ratio. The third term denotes the nuclear Zeeman effect where *γ*_*n*_ is the ^13^C nuclear spin gyromagnetic ratio. And the last term describes the hyperfine interaction between the electron spin and the nuclear spin of ^13^C atom.

Using a permanent magnet, an external magnetic field *B*_*z*_ is applied parallel to the NV axis. Hence, 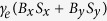 and 
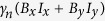
 are removed. Under the condition of weak magnetic field strength, the difference between the zero-field splitting *D* = 2.87 GHz and the electronic Zeeman splitting is much larger than the hyperfine interaction. In this situation, the electron-nuclear-spin flip-flop processes induced by the hyperfine interaction are sufficiently suppressed. Therefore, this allows for the secular approximation^56^,^57^,^58^, and the *S*_*x*_*I*_*x*_ and *S*_*y*_*I*_*y*_ terms can be neglected. In other words, for the weak external magnetic field along the NV axis only the longitudinal hyperfine interaction needs to be taken into account, and the ground-state manifold of the NV center coupled with a proximal ^13^C nuclear spin is described by the Hamiltonian


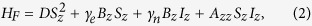


where


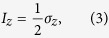



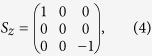


and *σ*_*z*_ is the Pauli-*z* operator.

#### The Hamiltonian under measurement

A microwave driving is utilized to perform the measurement, as shown in Fig. 1(b). This microwave pulse drives the transition between 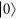 and 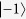. The driving frequency is set to be resonant with the transition between 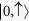 and 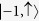, and meanwhile largely detuned from that between 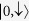 and 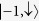. In this way, the transition between 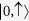 and 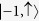 can be induced selectively. Thus the system evolves under the whole Hamiltonian *H*_*M*_ = *H*_*F*_ + *H*_*I*_, where the interaction Hamiltonian is


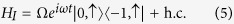


In order to analytically calculate the quantum dynamics under the influence of *H*_*M*_, we transform to the rotating frame defined by the transformation 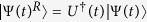, where *U*(*t*) = exp(−*iH*_*F*_*t*), 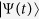 and 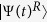 are the wave functions in the static and rotating frames respectively. Therefore, using equation (23), the Hamiltonian under the rotating frame can be obtained, i.e.,


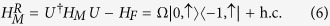


### Dynamic Evolution

Now, let us demonstrate the quantum dynamics of the whole system in a full cycle, which includes a free evolution followed by a measurement process. The electron spin is initially prepared in its ground state, i.e.,


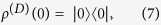


and the nuclear spin is in an arbitrary state


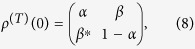


which is spanned by the basis 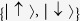. Thus, the initial state of the total system is 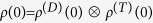.

In the free evolution, the total system evolves under its free Hamiltonian *H*_*F*_ for a time interval Δ*t*_*f*_, which is described by the evolution operator


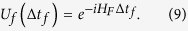


Apparently, without the driving, the evolution operator of the total system is diagonal. At the end of free evolution, the state of the total system becomes 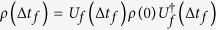. Afterwards, a microwave pulse is used to drive the transition between 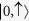 and 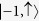. The total system evolves under the Hamiltonian 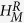 for a time interval Δ*t*_*m*_. Having transformed to the rotating frame, a time-independent Hamiltonian is obtained and the corresponding evolution operator is


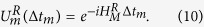


After a cycle with duration *τ* = Δ*t*_*f*_   + Δ*t*_*m*_, the state of the whole system is


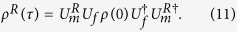


Utilizing equation (24), the final state of the whole system in the static frame reads


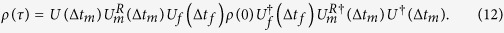


By partially tracing over the degree of the electron spin, the final state of the nuclear spin 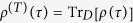 reads


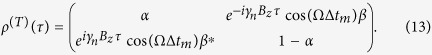


On the other hand, the initial state of the nuclear spin can be decomposed into its eigenbasis as^13^


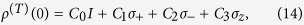


where *C*_0_ = 1/2, *C*_1_ = *β*, *C*_2_ = β^*^, *C*_3_ = (2*α* − 1)/2, *I* is the identity operator, 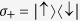 and 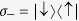 are the raising and lowering operators respectively. After the first cycle, the nuclear spin is in the state


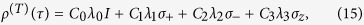


where the eigenvalues are


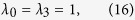



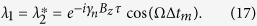


After *N* cycles, the nuclear-spin qubit evolves into the state


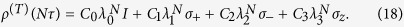


Here, *λ*_0_ = *λ*_3_ = 1 are related to fixed points^45^ independent of all parameters. However, *λ*_1_ and *λ*_2_ are modulated by the parameters (Ω, *B*_*z*_, Δ*t*_*m*_, Δ*t*_*f*_). By adjusting these parameters, the quantum Zeno and Zeno-like effects can be observed.

Hereafter, by analyzing the dependence of the eigenvalues on the parameters, we demonstrate the existence of quantum Zeno and Zeno-like effects.

#### Quantum Zeno-like effect

In equation (15), the eigenvalues *λ*_0_ = *λ*_3_ = 1 mean that 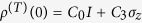 are the fixed points independent of the combination of parameters (Ω, *B*_*z*_, Δ*t*_*m*_, Δ*t*_*f*_) after repeated measurements. To be specific, if the initial state of the nuclear spin is of diagonal form, the state will not be changed and thus is preserved. This is a QZLE on the nuclear spin, similar to that in ref. 13.

On the other hand, the *σ*_+_ and *σ*_−_ components of the initial nuclear-spin state are exponentially suppressed, when 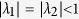 with appropriate parameters (Ω, *B*_*z*_, Δ*t*_*m*_, Δ*t*_*f*_). Therefore, the following process 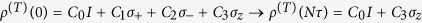 is achieved by sufficiently-many measurements.

Furthermore, the existence of *λ*_3_ = 1 preserves the polarization of the nuclear spin. If *α* is 1 or 0, 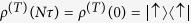 or 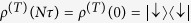 can be obtained. Thus, the polarization of the ^13^C nuclear spin near the NV center is frozen. It may be a potential way to preserve the polarization of ^13^C nuclear spin against its hyperfine interaction with electron spin.

#### Quantum Zeno effect

For the eigenvalues *λ*_1_ and *λ*_2_, we consider both the ideal situation when Δ*t*_*f*_, Δ*t*_*m*_→0 and the realistic situation of finite Δ*t*_*f*_ and Δ*t*_*m*_.

In the ideal situation, equation (17) is simplified as


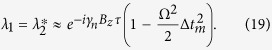


From equation (18), we learn that the eigenvectors *σ*_+_ and *σ*_−_ contribute to the off-diagonal part of *ρ*^(*T*)^(N*τ*). When Δ*t*_*f*_ and Δ*t*_*m*_ are small enough, the eigenvalues 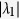 and 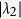 can be very close to unity. Furthermore, *γ*_*n*_*B*_*z*_*Nτ* = 2*nπ* is assumed, where *Nτ* = *T* is the fixed total evolution time and *n* is an integer. Because the eigenvalues 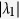 and 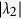 approach unity quadratically in the high-measurement-frequency limit, an arbitrary nuclear-spin state is exactly preserved by infinitely-frequent measurements. Here, the conventional QZE is recovered.

**Figure 2:**
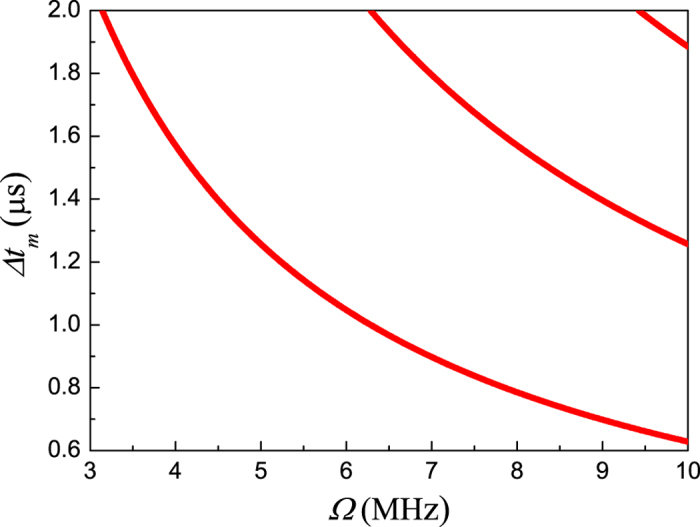
Measurement conditions for the QZLE. The QZLE will occur at the specified locations in the parameter space (Ω, Δ*t*_*m*_). The points are determined by equation (20) with *n*_1_ = 1, 2, 3.

On the other hand, consider the realistic condition in an NV center, e.g. non-vanishing Δ*t*_*m*_ due to a finite pulse width. When the Rabi frequency and the pulse width are chosen to meet the following requirement


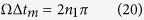


with *n*_1_ being positive integer, *λ*_1_ = *λ*_2_ = 1 can be obtained. In this case, arbitrary initial state is the Zeno-like fixed point which depends on the appropriate choice of parameters (Ω, *B*_*z*_, Δ*t*_*m*_, Δ*t*_*f*_). In other words, under certain measurement conditions, the QZLE is observed. Figure 2 shows the locations where QZLE will occur in the parameter space of (Ω, Δ*t*_*m*_). In the case of 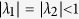, after repeated measurements the elements of *σ*_+_ and *σ*_−_ in *ρ*^(*T*)^(*T*) may disappear due to accumulated loss. However, by tuning parameters to the vicinity of the points (Ω, Δ*t*_*m*_) given in equation (20), equation (17) can be expanded to the second order of Δ*t*_*m*_ as


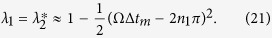


Here, the conventional QZE happens in the neighbourhood of the QZLE points. In other words, the QZE occurs for a series of parameter combinations corresponding to finite-frequency measurements with finite coupling strengths. Since the eigenvalues *λ*_1_ and *λ*_2_ approach unity quadratically under the repeated measurements, arbitrary nuclear-spin state is exactly preserved by finitely-frequent measurements, even though it is affected by the free evolution.

## Discussion

The conventional QZE and QZLE are demonstrated in a ^13^C nuclear spin around an NV center by controlling the electron spin. Both of the QZE and QZLE can be observed by modulating the Rabi frequency, and the magnetic field, and the free-evolution time, and the pulse width. Our numerical calculation properly shows that for suitable parameters there exist both the QZE and QZLE in an NV-center system under the experimental condition. Consequently, the conventional QZE and QZLE are obtained with finite-frequency imperfect measurements.

In order to put our experimental proposal into practice, the secular approximation should be valid, i.e. the applied magnetic field strength should not be too strong^56^. As a consequence, the magnetic field strength *B*_*z*_ could be less than 200 G. Additionally, due to the resonance condition, the driving frequency *ω* equals to the level spacing between 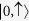 and 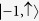, i.e., 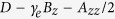. At the same time, the level spacing *ω*_1_ between 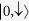 and 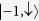 is 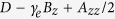. To selectively only induce the transition between 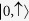 and 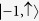, the large-detuning condition 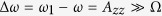 should be fulfilled. Since the hyperfine coupling between the electron spin and a ^13^C nuclear spin in the first coordination shell is known to be 130 MHz^51^,^59^, the Rabi frequency Ω can be no more than 10 MHz. Furthermore, the initialization of the NV center will take approximately ~140 ns^42^, and Δ*t*_*m*_ and Δ*t*_*f*_ are on a time scale about 2 *μs*. Thus, a single cycle process will take about 5 *μs*. The intrinsic dephasing time of the ^13^C nuclear spin *T*_2*n*_ was observed as around one second^41^. To ignore the decoherence effect induced by the environment, we restrict the total experiment time as 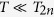, i.e., the total experiment time *T* is chosen as 100 ms. In this case, we can demonstrate the quantum Zeno and Zeno-like effects for roughly 2 × 10^4^ cycles.

On the other hand, due to the presence of the nitrogen nucleus, ^14^N (*I* = 1) or ^15^N (*I* = 1/2), and the ^13^C nuclear spin bath, the dephasing time of the electron spin is 58 *μs*^39^. The duration *τ* = Δ*t*_*f*_ + Δ*t*_*m*_ of a cycle is smaller than the dephasing time of the electron spin by one order. Because the hyperfine coupling between the electron spin and the nitrogen nucleus *A*_*N*_ < 4 MHz^50^ is much smaller than *A*_*zz*_, the dephasing effect induced by the nitrogen nucleus can be neglected. The dipole-dipole interactions between the electron spin and the other ^13^C nuclear spins are too weak. Therefore, the dephasing effect induced by all nuclear spins can also be neglected.

Last but not the least, the nuclear spin bath induces the dephasing of the ^13^C nuclear spin. Because the total experiment time is sufficiently short and the magnetic dipole-dipole interactions between the ^13^C nuclear spin and the other nuclear spins are weak enough^60^, the dephasing effect induced by the spin bath can be neglected. Meanwhile, after every measurement, we decouple the electron and ^13^C nuclear spins and initialize the electron spin in its ground state 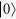
42,54 without perturbing the ^13^C nuclear spin. In this process, the nuclear spin is supposed to be completely isolated from the environment^42^,^54^. Therefore, the nuclear spin hardly evolves during this process.

In conclusion, as shown in Fig. 2, our numerical calculation properly indicates that under practical conditions we can demonstrate the conventional QZE and QZLE in ^13^C nuclear spin around the NV center with finite-frequency imperfect measurements.

## Methods

### The rotating frame

Since the original Hamiltonian in the measurement process *H*_*M*_ is time-dependent, the whole system is transformed to a rotating frame defined by the transformation 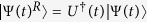, where 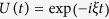, 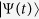 and 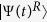 are respectively the wave functions in the static and rotating frames. Now, we derive the relationship between the Hamiltonian in the static frame *H*_*M*_ and the Hamiltonian in the rotating frame 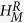. Because the time evolution of 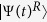 still fulfills the Schrodinger equation in the rotating frame, i.e.


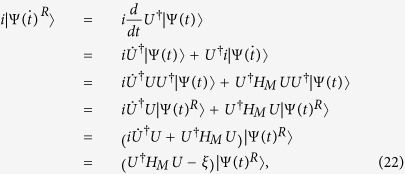


the effective Hamiltonian in the rotating frame reads


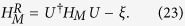


Correspondingly, the relationship between the density matrix in the static frame *ρ*(*t*) and the density matrix in the rotating frame *ρ*^*R*^(*t*) is


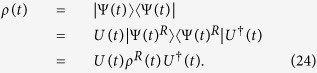


## Additional Information

**How to cite this article**: Qiu, J. *et al.* Quantum Zeno and Zeno-like effects in nitrogen vacancy centers. *Sci. Rep.* **5**, 17615; doi: 10.1038/srep17615 (2015).
